# ReAlignerV: Web-based genomic alignment tool with high specificity and robustness estimated by species-specific insertion sequences

**DOI:** 10.1186/1471-2105-9-112

**Published:** 2008-02-22

**Authors:** Hisakazu Iwana, Yukio Hori, Kensuke Matsumoto, Koji Murao, Toshihiko Ishida

**Affiliations:** 1 Life Science Research Center, Kagawa University, Ikenobe 1750-1, Miki-cho, Kita-gun, Kagawa, 761-0793, Japan; 2 Faculty of Medicine, Kagawa University, Ikenobe 1750-1, Miki-cho, Kita-gun, Kagawa, 761-0793, Japan; 3 Information Technology Center, Saiwai-cho Branch Office, Kagawa University, 2-1 Saiwai-cho, Takamatsu-shi, Kagawa, 760-8521 Japan; 4 Division of Endocrinology and Metabolism, Department of Internal Medicine, Faculty of Medicine, Kagawa University, Ikenobe 1750-1, Miki-cho, Kita-gun, Kagawa, 761-0793, Japan

## Abstract

**Background:**

Detecting conserved noncoding sequences (CNSs) across species highlights the functional elements. Alignment procedures combined with computational prediction of transcription factor binding sites (TFBSs) can narrow down key regulatory elements. Repeat masking processes are often performed before alignment to mask insertion sequences such as transposable elements (TEs). However, recently such TEs have been reported to influence the gene regulatory network evolution. Therefore, an alignment approach that is robust to TE insertions is meaningful for finding novel conserved TFBSs in TEs.

**Results:**

We constructed a web server 'ReAlignerV' for complex alignment of genomic sequences. ReAlignerV returns ladder-like schematic alignments that integrate predicted TFBSs and the location of TEs. It also provides pair-wise alignments in which the predicted TFBS sites and their names are shown alongside each sequence. Furthermore, we evaluated false positive aligned sites by focusing on the species-specific TEs (SSTEs), and found that ReAlignerV has a higher specificity and robustness to insertions for sequences having more than 20% TE content, compared to LAGAN, AVID, MAVID and BLASTZ.

**Conclusion:**

ReAlignerV can be applied successfully to TE-insertion-rich sequences without prior repeat masking, and this increases the chances of finding regulatory sequences hidden in TEs, which are important sources of the regulatory network evolution. ReAlignerV can be accessed through and downloaded from .

## Background

Cross-species comparisons of genome sequences have provided an efficient means of identifying conserved functional elements. Alignment procedures of related species are the mainstay for comparative genomics [1]. Identification of CNSs followed by functional motif discovery has successfully revealed its power with regard to both yeast species [2,3] and mammals [4]. The combination of alignment-based CNS detection and TFBS prediction is also recognized as one of the promising approaches. Today, a wide variety of methods (for example, rVISTA [5], CONREAL [6], ConSite [7], etc.) are publicly available through the web. For the alignment, rVISTA utilizes BLASTZ [8], whereas CONREAL adopts a greedy strategy by allowing users to choose from LAGAN [9], MAVID [10] and/or BLASTZ.

The aligners described above adopt heuristic index-based approaches to decrease processing time and memory usage. In order to obtain sufficiently long alignments, they utilize a chaining strategy based on the highly similar aligned sequences which are called anchors or seeds. In addition, repeat masking processes are, in many cases, a prerequisite in advance of alignment. These approaches indeed fulfill a demand to deal quickly with genome-wide long sequences and to obtain sufficiently long aligned blocks. However, this genome-wide resolution may not always be an optimal answer; for instance, to the researchers specifically focusing on noncoding regions stretching no more than several tens of kbs or those using alignment tools to construct the best short list in advance of laborious experimental validations.

One reason is that the demand to obtain as-long-as-possible alignments tends to result in over-extended aligned stretches, which reduces the sharpness of the boundary of short conserved sequences. Another is the possibility that repeat masking processes, that are in many cases performed as a preprocess, can hide the important functional elements that are embedded in the TE insertion sequences. In fact, TE insertions are now recognized as an important source of evolutionary processes of regulatory elements. The majority of duplicated repressor element 1 (RE1) sequences have been reported to exist within TEs [11]. Experimental evidence has shown that an enhancer element in the CNSs, which is also located within a short interspersed nuclear element (SINE) family, is active in some extant tetrapods [12]. A systematic analysis has also shown that ~2.5% of experimentally validated *cis*-elements are overlapped with TEs and those *cis*-elements correspond to ~4.5% genes examined [13].

Consequently, in this report, we describe the features of ReAlignerV with emphasis on robustness to TE insertions in aligning genomic sequences. To estimate the robustness to TE insertions we focused on SSTEs, i.e. TEs specific to human or rodent. Since the SSTEs have been inserted into the genomes after the divergence of human and rodents (mouse and rat) from their common ancestor, if the SSTE regions are aligned to the orthologous counterpart sequence between human and rodents, the counterpart nucleotide sites corresponding to the SSTEs are expected to be gaps in the alignment. Therefore, we have deemed SSTEs to be negative control probes and evaluated the specificity of the aligners using 1,490 trios (human, mouse and rat) of orthologous 8-kb noncoding sequences immediately upstream of the protein-coding sequences. We applied this evaluation procedure to ReAlignerV, BLASTZ, LAGAN, MAVID and AVID [14].

## Implementation

### Inputs

**Figure 1:**
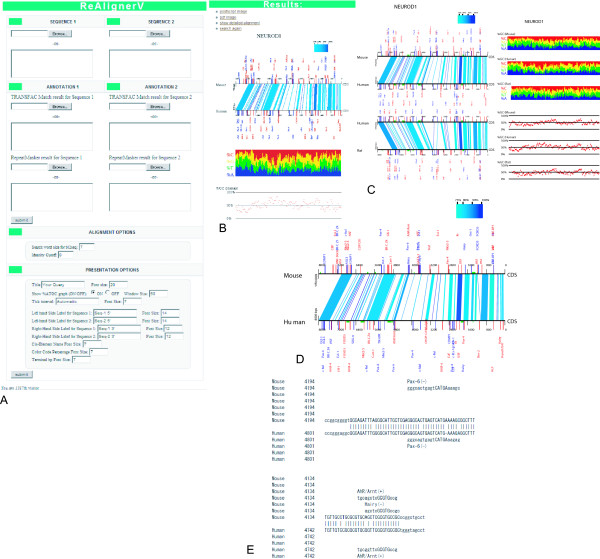
**An overview of the ReAlignerV web service**. Through the input webpage (A), users can input the query sequences, their annotation data and parameters. The ReAlignerV server returns the schematic alignments for 2-species (B) or 3-species (C) comparisons with or without graphs of the GC and ATGC contents. In the results, the aligned blocks are shown by blue-color-coded boxes. The results pages still hold the windows for annotations so that users can modify the results presentation interactively through this web service after the alignment procedure. Once the alignment computation has been done, the annotation processes are quick. An enlarged snapshot (D) of the schematic alignment shows how the predicted TFBS and TEs are integrated and the locations of predicted TFBS are indicated by red and blue ticks with their names in the same colors. Green horizontal bars along the alignment lines designate the positions of TEs (D). From both the 2- and 3-species ReAlignerV results pages, users can jump to a pair-wise alignment (E), where the predicted TFBS sequences are also shown alongside each aligned sequence.

A pair of FASTA format nucleotide sequences are minimally required as input for the pair-wise alignment (Figure 1A). The web server assumes that the right-hand side ends of these sequences are single anchors that correspond to each other. For instance, either right end of the two sequences may correspond to the first nucleotide immediately upstream of the start codon of each sequence. ReAlignerV can also accept masked sequences in which the masked nucleotides are indicated by Xs. Unless users have an interest in TEs, repeat-masked sequences are preferable for accurate alignments. It should be noted that the approach of masking only SSTEs could be useful in reducing noise caused by SSTEs without sacrificing the possibility of finding any functional elements located in old TEs. For integrated presentations, the results of TRANSFAC(R) [15,16] database search program Match™ [17,18] and RepeatMasker [19] can be used as the annotation input source for each sequence (Figure 1A).

### Aligning two sequences

To align two sequences, users can select the '2-species ReAlignerV' from the ReAlingerV homepage, and either paste the FASTA format sequences or upload the sequence files. After submission, the results page is automatically refreshed every few seconds until the resulting alignment is presented. Before submission, users can adjust the alignment parameters as necessary. Input 'sequence 1' is always presented as the lower black line of the schematic alignment presentation. Below the alignment, the GC and ATGC contents for sequence 1 are shown by the specified scanning window length (Figure 1B), if the '%ATGC graph option' is toggled on (default). Users can modify the resultant annotation and appearance of presentations interactively through the web after the alignment procedure has finished. Since this modification is performed quickly, we recommend that users perform the alignment procedure in advance.

### 3-species ReAlignerV

As an option, users can conduct a three-species comparison, which consists of two runs of pair-wise alignments, *i.e*. sequence 1 vs. sequence 2 and sequence 1 vs. sequence 3, rather than multiple alignment. The resultant two pair-wise alignments are integrated into one schematic presentation together with the annotations to the three input sequences. The graphs of the GC and ATGC contents of the three sequences are also provided. Sequence 1 is always presented as two lines in the middle of the two alignments (Figure 1C).

### Adding TFBS annotation

As TFBS prediction information, the TRANSFAC database Match program search results for each input sequence can be integrated into the resultant alignment. After completing the Match search [16] on the TRANSFAC database, the user can copy the tabular portion of the Match results and paste this into the window of the ReAlignerV 'TRANSFAC Match result' (Figure 1A). Then, after clicking the submit button, users will soon see the integrated presentation, in which each predicted TFBS location is indicated either by a red tick (for a same strand hit) or by a blue one (for an opposite strand hit), with the TFBS name presented alongside (Figure 1B, C and 1D).

### Adding RepeatMasker annotation

An annotation file from RepeatMasker, with the extension '.out', can be integrated into the alignments. Users can copy and paste the tabular part of the RepeatMasker annotation results into the 'RepeatMasker result' window in ReAlignerV. After submitting this data, the regions of repeat sequences are shown by green bars along the horizontal black lines (Figure 1D illustrates this in detail).

### Detailed alignment presentation

In addition to the schematic alignment, pair-wise alignments are available for every aligned block through this web service by clicking on the 'Detailed alignment' in the results page. The important feature of this 'Detailed alignment' is that the predicted TFBS sequences, together with the respective TFBS names, are shown along the aligned sequences, which enables users specifically to pin down the conserved TFBS candidate sites (Figure 1E). In this alignment, each aligned sequence has a 10-nt overhang stretch of sequence extending from either side of the aligned block. The overhang stretch is shown in lower-case letters, while the aligned sequences are shown in upper-case. This overhang is useful in examining an alignment in which a predicted TFBS motif match extends across the border of an aligned block. When TRANSFAC Match search results are used, the TFBS motif core which has less degeneracy is indicated by upper-case letters and the rest of the motif is shown by lower-case letters, which follows the representation of the Transfac database records.

### Presentation options

Users can adjust a series of presentation options such as the tick interval, the contents of labels and their font sizes. Since the result image is provided in postscript^® ^format, which can maintain the original resolution, it is readily available for publication. Users can alternatively download the PDF format, or choose an email transfer option.

### Pair-wise alignment algorithm

**Figure 2:**
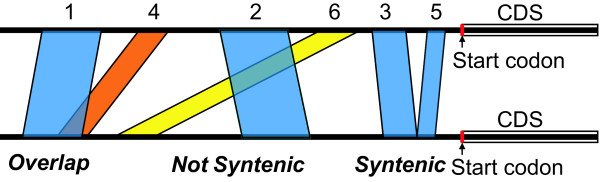
**Schematic explanation of the re-aligning processes**. ReAlignerV retrieves the local alignments that are produced by bl2seq in decreasing order of the bit scores. Colored blocks represent the same-direction locally aligned portions. The number above each block indicates the order of the degree of bit score. ReAlignerV adopts blue blocks and discards red and yellow ones to construct the resultant alignment. The scheme illustrated here shows the case where the noncoding sequences immediately upstream of the start codon were used to assess the specificity and robustness to TEs for the 1,490 8-kb trio orthologs.

This web server adopts the alignment method based on REALIGNER [20,21]. In brief, this method initially searches the two input sequences for locally aligned sequences using bl2seq [22]. To align short conserved blocks in the noncoding sequences appropriately, the parameter set of bl2seq is adjusted, so that word size = 7 (default) and mismatch penalty = -2 (preset). From the resultant alignments, only the same strand hits are retrieved. In decreasing order of the bit score for each local alignment, the following two steps are repeated (see Figure 2). First, when two alignments overlap, the program removes the one with the lower bit score and retains the other. Second, when two alignments are not syntenic, the alignment with the lower bit score is removed and the other is retained. If the bit scores being compared are equal, first the longer hit-stretch and then the more downstream alignment has the higher priority.

### Downloadable version of ReAlingerV

A command-line stand-alone version of ReAlignerV can be obtained from [21], together with instructions and the test data set. This downloadable script contains all the functions that are provided through the web service for the 2-species ReAlignerV.

## Results

### Orthologous sequence set for comparison of aligners

We downloaded the reference genome sequence (RefSeq) and annotation files [23,24] of human (NCBI Build 36.1), mouse (NCBI Build 36.1) and rat (RGSC v3.4) from NCBI, and surveyed the features of all the nuclear protein-coding genes for the three species. We manually corrected the annotation of these genes according to Entrez GENE[25]. Then, we selected every human gene with a HUGO (Human Genome Organization)-approved official gene name and symbol [26,27]. From these genes, in order to collect the orthologs among human, mouse and rat, we retrieved the genes with an identical gene symbol in the three species, amounting to 1,899 gene trios. To reduce the false positive assignment of orthologs, we selected genes that are annotated stably across the three species rather than adopting the approach of mere pair-wise matches. This would contribute to obtaining a robust dataset based on which we could assess specificity and significance of the alignment methods. This method of ortholog assignment has the advantage of stringently avoiding contamination of paralogs, because these genes are annotated based not only on sequence homology but also on evidence from functional and physiological examinations. We further selected the ortholog trio genes by confirming the following four conditions for all three species: i) the 9-kb sequence upstream of the translation start site does not include any annotated genic regions, ii) the gene does not overlap with another gene, iii) the gene is not completely included by another gene, and iv) the gene does not include another gene completely within it. Finally we obtained 1,490 ortholog trio genes, and for these trio genes, we excised the 8-kb stretch upstream of the translation start site. The 1-kb margin is set to avoid contamination of the 3' or 5' control sequences which belong to the flanking genic region.

### Species-specific TE detection

We executed RepeatMasker to identify the SSTE insertions. For all the 8 kb stretches of 1,490 human sequences, the "primspec" option was used to detect only the primate-specific TEs, and "rodspec" option was used for all the mouse and rat 8-kb upstream sequences. Other RepeatMasker options used were "gccalc", to follow the actual GC contents of the input sequences, and "norna", not to mask putative small RNA genes that may be encoded in the upstream region.

### ReAlignerV alignments for trios

We conducted alignment procedures for the 1,490 trios of orthologous sequences using the aligner of ReAlingerV in a local machine setting. The conditions used were the same as the default settings of the ReAlignerV web service.

### BLASTZ alignments for trios

We downloaded BLASTZ packaged in the PiPMaker distribution (beta-version) pipmaker-2005-10-26-01 [28], and used the parameter set with Y = 3400, H = 0, W = 5, B = 0, K = 1600, C = 2, and P = 0. These parameter settings are the same as those used when "Advanced PipMaker" is run from its web server with the options 'Search on strand', 'Chaining', and 'High sensitivity and low time limit' toggled on. We used this version of BLASTZ for the 1,490 trio ortholog set without prior repeat masking.

### LAGAN alignments for trios

We downloaded the lagan12 source code [29]. After installing it according to the instructions, we performed pair-wise global alignment for the 1,490 trio ortholog set by executing lagan.pl with default settings and without using repeat-masked files.

### MAVID alignments for trios

We downloaded MAVID (mavid-package-2.0.4) [30] after registering for an academic license. According to the instructions of MAVID, we downloaded both CLUSTALW (clustalw1.83) [31,32] and fastDNAml, (fastDNAml_1.2.2) [33,34]. After installing them correctly, we executed mavid.pl with the option "-r" to refine final alignments and with the other options as the default settings. To obtain alignments in which TE insertion sequences are not repeat-masked, we prepared the repeat-mask sequence files identical to the files of input sequences and these pairs of identical files were processed by MAVID.

### AVID alignments for trios

After having obtained authorization, we downloaded and installed AVID (version 2.1 build 0) [35] according to the instructions. We executed AVID to process the trio-ortholog data set with the option "-nm = both" so as not to mask the TE insertions, and with the other options as the defaults.

### Statistical analysis

We use three statistics, namely specificity (*f*), alignment cover rate (ACR) and significance index (*z*), to assess the robustness of the alignment procedures to TE insertions. To investigate the relationship of these statistics to the content of the SSTEs, we applied ranges based on the number of SSTE sites for the human. For the human statistics, we summed the number of sites in both the human-mouse and human-rat alignments. The following notations are used for each range in the human sequences.

*t*_[range] _= the total number of sites that were examined,

*a*_[range] _= the total number of aligned sites,

*i*_[range] _= the number of observed SSTE insertion sites,

*m*_[range] _= the number of aligned sites which are located in the SSTEs, *i.e*. misaligned sites.

Taking *m *as the number of false positives and *i *- *m *as the number of the true negatives, the specificity (*f*) against the SSTE is computed as,

*f*_[range] _= (*i*_[range] _- *m*_[range]_)/*i*_[range]_.

To estimate how extensively an aligner makes alignments, we define the ACR as,

ACR_[range] _= *a*_[range]_/*t*_[range]_.

The probability *p *of an aligned site being located within the SSTE sites by chance is given as,

*p*_[range] _= *i*_[range]_/*t *_[range]_.

Then, for each range, we assume that *m *(*i.e*. the number of the false positives) follows the binominal distribution with a probability *p *and the number of trials *a*. Thus, the standard deviation is computed as,

*s*_[range] _= {*a*_[range] _*p*_[range] _(1 - *p*_[range]_)}^1/2^,

and its expectation (*e*) as,

*e*_[range] _= *a*_[range] _*p*_[*range*]_.

We define the significance index (z) as,

*z *= (*e*_[range] _- *m*_[range]_)/*s*_[range]_.

### High specificity of ReAlignerV against SSTEs, but low ACR

**Figure 3:**
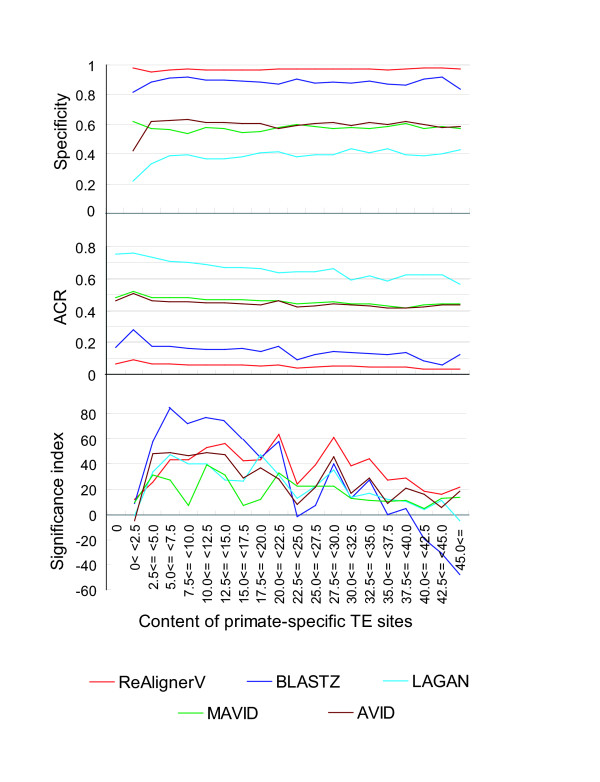
**Comparison of 5 aligners using the 1,490 8-kb trio ortholog set**. Specificity, measured by SSTE insertions (A), ACR (B) and the significance index (C) are shown for each range of the content of primate-specific TE insertions within the human 8-kb noncoding upstream sequences. ReAlignerV shows the best specificity across all ranges, but scores the lowest values for ACR. The significance index, which takes both the specificity and ACR into account, reveals that ReAlingerV has the highest robustness in the ranges where TE content is 20% or higher.

After completing 1,490 8-kb human-mouse and human-rat ortholog alignments, ReAlignerV showed the highest specificity across all ranges of the SSTE insertion content, and its specificity values remained close to 1 even in the ranges surpassing 45% SSTE content (Figure 3A). Since there is often a trade-off between specificity and sensitivity, it is likely that an aligner that extensively stretches alignments tends to have a lower specificity. Since we do not know the true alignment for an actual noncoding sequence dataset such as the one we have used, the sensitivity is theoretically immeasurable. Thus, instead we compared the alignment cover rate (*i.e*. ACR) for each aligner to assess how extensively it makes alignments. The results show that LAGAN performed best by making extensive alignments, followed by AVID and MAVID, while ReAlignerV yielded modest lengths of aligned stretches (Figure 3B). The order of the goodness of specificity is just the reverse of that of the ACR. This trade-off trend between ACR and specificity is consistent with the results of benchmarking studies conducted on simulated datasets [36,37].

### Robustness to TE insertions

To evaluate the robustness to TE insertions, it is necessary to assess whether the gain in specificity compensates for the drop in ACR and vice versa. For this assessment, we adopted the significance index (*z*) to measure specifically how an aligner avoids SSTE sites and extends the alignments. The significance index of each aligner tends to decline according to the increase in proportion of SSTEs (Figure 3C). This can be explained by reasoning that the SSTE insertions cause noise that disturbs the alignment procedure. The significance index for ReAlignerV is the best in the ranges with more than 20% SSTE content. The significance index for BLASTZ is the best in the ranges in which the SSTE content is less than 20%, and it drops when exceeding 20% SSTE content. These results suggest that ReAlignerV has an advantage in constructing alignments for insertion-rich sequences without prior repeat masking processes.

### Interpretation of the aligner comparison considering algorithmic aspects

The current comparison of aligners was performed under the conditions whereby alignments are constructed by i) several kb lengths of orthologous upstream sequences ii) of human and rodent, iii) without repeat masking, and iv) focusing on the search for conserved TFBSs around promoter regions. The condition of no repeat masking can particularly affect the performance of BLASTZ which is presupposed to deal with SSTE-masked sequences in its original setup. Therefore, the drop in significance index in the ranges of high SSTE contents is attributable to the unintended use, and the highest significance index in the ranges of low SSTE contents is more indicative of the original worth of BLASTZ. On the other hand, this result indicates that the evaluation scheme is reliable. Since AVID and MAVID also utilize the repeat sequence information for the anchor selection step, the condition of not having repeat sequence information available could negatively affect the performances. LAGAN, AVID and MAVID adopt complex approaches in which the Needleman-Wunsch algorithm is compositely used, for example, in the case where an inter-anchor region to be aligned is sufficiently short, and furthermore the three aligners and BLASTZ adopt a recursive call to the alignment procedure, which leads to high ACRs. Compared to these, ReAlignerV uses a straightforward strategy to set the single anchor at the last nucleotide of each query sequence and performs no recursive alignment process after the seed extension. Despite such a simple approach, ReAlignerV shows a good agreement between specificity and ACR, indicated by the high significance index for the dataset used. The expected advantage of the eager alignment approaches may not always give the best significance particularly under the conditions described above that are suitable to search for conserved TFBS including TE-related ones around promoter regions.

### An example ReAlignerV usage for experimental validation

**Figure 4:**
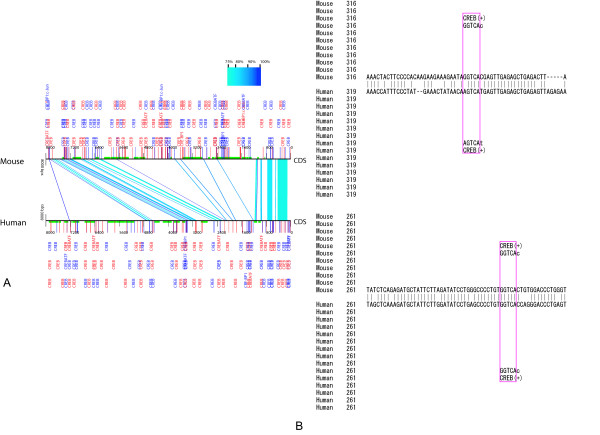
**CRE prediction by ReAlignerV followed by experimental validation**. The 8-kb genomic sequences upstream of the translation start sites of human and mouse CCL3 genes were aligned, and the alignments were integrated with results from RepeatMasker and TRANSFAC Match searches by ReAlignerV. In this prediction, only CRE sites were searched for by TRANSFAC Match (A), and two conserved CRE sites, shown in red squares (B), were found to be functional by an experimental study.

To identify the cAMP response element (CRE) candidates located in the upstream regions of mouse chemokine (C-C motif) ligand 3 (CCL3) or the MIP1a gene, we used the locally installed ReAlignerV together with the results of TRANSFAC Match and RepeatMasker searches. The integrated presentation of the human-mouse sequence comparison of the CCL3 8-kb genomic sequences upstream of the translation start site indicated several conserved CRE candidates (Figure 4A and 4B). For this analysis, we generated a user-defined factor matrix set including 11 CRE motif matrices using Match Profiler [38] and the TRANSFAC Professional (version 10.1) database. We conducted a Match search on this matrix set under a less stringent condition where the false negative rate = 10 %, and this predicted 131 CRE sites for human and 155 for mouse (Figure 4A). From this large number of predicted sites, we first focused on the conserved CRE sites and identified six potential CRE sites that were located in the corresponding conserved blocks with the same motif direction by examining the detailed alignment of ReAlignerV. Of the six, four conserved predicted CRE sites that resided within 1-kb vicinity upstream of the human and mouse transcription start site were prioritized for the experimental validation. From these, we tested whether the four predicted CRE sites were functional in human adult T-cell leukemia (ATL) cell lines by the reporter gene assay, which resulted in two of the four predicted elements being functional (Matsumoto *et al*. unpublished data). This result indicates that ReAlignerV is useful in efficiently narrowing down the TFBS candidates in advance of experimental studies. In our analysis, we did not find any conserved TFBS candidates that overlapped with TEs by RepeatMasker (version Open-3.1.5, with -s option, RepBase version 11.02). Besides the conserved sites, however, we found that 11 predicted CRE sites for human were located in primate-specific Alu sequences and 2 sites for mouse in a rodent-specific B2 (SINE) sequence. These CRE sites could be SSTE-mediated TFBS candidates worth further investigation as to whether they could affect the linage-specific transcriptional control.

## Conclusion

We have shown that ReAlignerV has a high degree of specificity and robustness regarding TE insertions in aligning TE-rich noncoding sequences. Thus, ReAlignerV is applicable to aligning insertion-rich sequences without prior repeat masking. This feature could present the possibility of detecting novel functional elements hidden in TE insertions. ReAlignerV provides intuitively understandable presentations and a web-based interactive alignment annotation method, which helps identify a best short list of functional elements in advance of experimental validations. Future extensions to this work include i) smoother interconnections with TRANSFAC database searches and RepeatMasker, ii) wider acceptability of the other TFBS prediction methods, iii) focusing on 3' noncoding regions, and vi) speed- and scale-up of the aligner by reprogramming using a faster language.

## Availability and requirements

Project name: Research Program on Gene Regulatory Network

Project home page: 

Programming language: Perl

License: GNU General Public License

Any restrictions to use by non-academics: The restrictions specified by the organizations that maintain bl2seq, Match™, TRANSFAC(R) and RepeatMasker, apply to the use of the programs and databases.

## Authors' contributions

HI initiated this web server project, wrote the original source code, performed the comparison of the aligners on specificity and robustness, and wrote the manuscript for ReAlignerV. YH arranged the source code, constructed the web interface, and implemented it on the server. K Matsumoto, K Murao and TI performed all the experimental studies on the CCL3 regulatory elements. A limited part of their results was used as the example for actual usage of ReAlignerV, and they cooperatively designed the web service particularly from the viewpoint of an experimental research field.
